# Polymeric Electrospun Fibrous Dressings for Topical Co-delivery of Acyclovir and Omega-3 Fatty Acids

**DOI:** 10.3389/fbioe.2019.00390

**Published:** 2019-12-03

**Authors:** Tiago Costa, Artur Ribeiro, Raul Machado, Clarisse Ribeiro, Senentxu Lanceros-Mendez, Artur Cavaco-Paulo, Andreia Almeida, José das Neves, Marlene Lúcio, Teresa Viseu

**Affiliations:** ^1^Centre of Physics of Universities of Minho and Porto (CF-UM-UP), University of Minho, Braga, Portugal; ^2^Centre of Biological Engineering (CEB), University of Minho, Braga, Portugal; ^3^Centre of Molecular and Environmental Biology (CBMA), University of Minho, Braga, Portugal; ^4^Institute of Science and Innovation for Sustainability (IB-S), University of Minho, Braga, Portugal; ^5^Basque Center for Materials, Applications and Nanostructures (BCMaterials), UPV/EHU Science Park, Leioa, Spain; ^6^Basque Foundation for Science (Ikerbasque), Bilbao, Spain; ^7^Institute for Research and Innovation in Health (i3S), University of Porto, Porto, Portugal; ^8^Institute of Biomedical Engineering (INEB), University of Porto, Porto, Portugal; ^9^Institute of Biomedical Sciences Abel Salazar (ICBAS), University of Porto, Porto, Portugal

**Keywords:** acyclovir, omega-3 fatty acids, electrospinning, poly (ε-caprolactone), polymeric fibrous dressings

## Abstract

Herpetic infections caused by Herpes simplex virus (HSV) are among the most common human infections, affecting more than two quarters of the world's population. The standard treatment for orofacial herpes is the administration of antiviral drugs, mainly acyclovir (ACV). However, current products are mostly based on semisolid formulations that have limited ability to promote drug skin penetration and tend to leak from the application site, thus showing reduced ability to sustain local drug residence. This work reports on the production of poly (ε-caprolactone) (PCL) fibrous matrices with ACV and omega-3 fatty acids (ω3) for application as dressings to the topical treatment of orofacial herpes. PCL fibrous matrices with the co-incorporated bioactive compounds were obtained by electrospinning and characterized regarding their morphology, chemical, physical, and mechanical properties. The potential use of the developed polymeric fibrous matrices for topical applications was evaluated by: (i) the release kinetics of the bioactive compounds; (ii) the occlusive factor of the fibrous mat; (iii) ACV skin permeation capacity; and (iv) the cytotoxicity in a keratinocyte cell line. PCL fibrous matrices loaded with the bioactive compounds presented a smooth morphology and a good balance between flexibility and hardness essential to be durable for handling, while having a desirable texture to be used comfortably. The fibrous mat also provided a sustained release of ACV during 96 h and improved the skin permeability of this drug (Kp = 0.00928 ± 0.000867 cm/h) presenting also high porosity (74%) and a water vapor transmission rate (WVTR) of 881 ± 91 g/m^2^day, essential to maintain moist and oxygen for faster healing of herpes lesions. Furthermore, cytotoxicity studies suggest that the fibrous mat are safe for topical application. Overall, the PCL based electrospun fibrous matrices with ACV and ω3 hereby described have the potential to be used as therapeutic bandage systems for the treatment of orofacial herpes.

## Introduction

Herpes are a group of infectious diseases very common in humans, caused by direct exposure to the *Herpesviridae* family viruses or through contact with objects contaminated with these viruses (Mustafa et al., 2016). About 130 species of *Herpesviridae* capable of infecting living beings have been identified but only eight of them are capable of infecting humans (*Human herpesvirus* or HHV) and cause clinical manifestations (Looker et al., 2015). The most common HHV viruses are Herpes simplex virus (HSV), in particular subtypes 1 and 2 (HSV-1 and HSV-2), varicella-zoster virus (HHV-3 or VZV), Epstein-Barr (HHV-4 or EBV) and cytomegalovirus (HHV-5 or CMV) (Grinde, 2013). Infections caused by HSV are one of the most common human diseases affecting 60–95% of the world's population (Jiang et al., 2016). These viral agents are responsible for a wide range of pathologies that range from simple, easily treatable genito-facial, and orofacial lesions (gingivostomatitis, herpes labialis and genital herpes) to more severe infections affecting the eyes (keratoconjunctivitis) and central nervous system (herpetic encephalopathy and meningitis) (Javad et al., 2014; Lopes et al., 2018).

**Figure 1:**
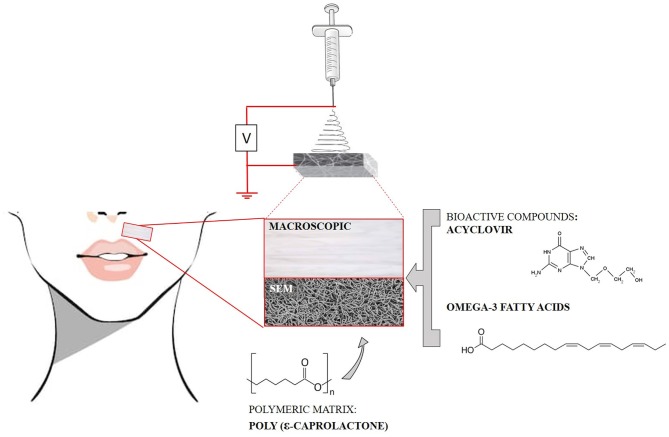
Schematic representation of the electrospinning process used for production of a fibrous system based on a polymeric matrix, containing ACV, and omega-3 fatty acids (ω3), for treatment of orofacial herpetic infections. A photo showing the macroscopic aspect of the polymeric matrix as well as its scanning emission microscopy (SEM) image are presented.

Given the high prevalence of these viral infections, the pharmaceutical industry has concentrated its efforts on the development of antiviral drugs. Currently the most widely used drug for the treatment of herpes is acyclovir (ACV), approved by the Food and Drug Administration (FDA) since 1982 (Elion, 1993; Durai, 2015; Mustafa et al., 2016). ACV (Figure 1) is a prodrug, analog of 2'-deoxyguanosine, known for its activity against HSV. Considering the incidence of herpes orofacial pathologies, topical administration of ACV would be the preferable and recommended route of administration. As compared with oral administration, topical administration of ACV leads to 10-fold higher concentration over the entire epidermis (Jain et al., 2011). However, this concentration fails to produce the desired therapeutic effect at the site of infection because of the low penetration of ACV in the basal epidermis (Lembo and Cavalli, 2010; Jain et al., 2011). Indeed, despite its effectiveness at viral target level, ACV has a number of handicaps, namely low lipophilia, low solubility in water, and low membrane permeation (0.12 × 10^−6^ to 2.0 × 10^−6^ cm/s) (Lopes et al., 2018), which limit the skin penetration of this drug and also its solubilization in the formulation vehicles. Consequently, ACV topical commercial formulations are based in supersaturated formulations of ACV (5 mg/g) that need to be applied 5 to 6 times per day for 4–6 days compromising patients' compliance to the therapy (Lembo and Cavalli, 2010; Szunerits et al., 2015; Lopes et al., 2018). Furthermore, therapeutic effective levels of ACV often fail in view of the numerous applications required to maintain efficacious levels of the drug at the site of application. It is thus evident the need to develop alternative systems that allow a better distribution of ACV and its topical delivery in a more convenient way to minimize therapeutic failure (Lopes et al., 2018).

In the last years, polymeric fibrous matrices have gained more interest in the academic and industrial fields, in particular in biomedical sciences, as drug delivery systems for the incorporation of drugs (Hu et al., 2015; Khodir et al., 2018) and other bioactive compounds (Hu et al., 2016; Pinese et al., 2018). These systems can be used for the sustained release of drugs and can improve their therapeutic performance. In this context, the incorporation of antiviral drugs, such as ACV, in polymeric fibrous matrices is foreseen to have a great potential for the treatment of infections caused by HSV-1 and HSV-2. Polyacrylonitrile (PAN) and polyvinylpyrrolidone (PVP) are two of the most used polymers for the incorporation of ACV and were used for the production of functional fibrous matrices for the transdermal delivery of this drug (Chen and Yu, 2010; Yu et al., 2010, 2011). Fiber mats composed by polylactic acid (PLLA) were also used for ACV delivery in the treatment of infections of the female reproductive tract (Ball et al., 2012). Moreover, Baskakova et al. reported the production of poly (ε-caprolactone) (PCL) fibrous matrices loaded with ACV for the treatment of ocular infections with the co-incorporation of other actives (ciprofloxacin and cyanocobalamin) (Baskakova et al., 2016).

In view of the successful attempts to load ACV in polymeric fibers, herein we propose the development of PCL fibrous matrices, prepared by electrospinning, to be used as dressings for the topical delivery of ACV and fish oils rich in omega-3 fatty acids (ω3). Hence, the fibrous system purposed aims the treatment of orofacial herpetic infections (due to ACV delivery) and the healing of the herpetic lesions (due to the fibrous dressing properties and ω3 delivery) (Figure 1).

Electrospinning was chosen as the preparation technique. Obtained fibers by this preparation process are versatile carriers for various therapeutic applications, since they allow the incorporation of several drugs and/or bioactive principles in high concentrations and, by altering the processing parameters of the electrospinning, it is possible to adapt the properties of the fibers for the intended application (Pillay et al., 2013). Furthermore, it is an inexpensive and easy-to-scale process. As for the composition of the fibers, PCL was selected because of its previous use for ACV loading and its high mechanical resistance, biodegradability, and biocompatibility thus making the fibrous matrices composed by this polymer suitable for topical administration. Despite PAN and PVP are the most used polymers for the incorporation of ACV, PCL has interesting advantages over these polymers, particularly in the context of a dressing for herpes orofacial lesions. In comparison with both polymers, PCL has better mechanical properties combining resistance with exceptional flexibility (Ulery et al., 2011). It is thus expected that this polymer will have a better performance maintaining the herpes dressing closer to the skin contour even under facial movements. However, the only ACV loaded PCL systems reported in the literature (Baskakova et al., 2016) were prepared with applications other than the orofacial herpetic lesions in mind. In this work we sought to develop a PCL polymeric system that could easily integrate a bandage for orofacial herpes treatment by co-delivery of ACV (with antiviral effect) and ω3 to help on tissue repair, minimizing the damage caused by the herpetic manifestation. Indeed, to minimize the effects of herpetic infections it may be appropriate to administrate other bioactives or natural compounds that have a beneficial effect in the healing process (Mustafa et al., 2016), such as ω3. These fatty acids (Figure 1) are able to increase the immune response and keep damaged tissues humid accelerating its growth and stimulating the healing process by promoting angiogenesis and epithelialization. Furthermore, incorporating the drug in a polymeric matrix to be used as a bandage system will provide a more convenient way for topical administration of a therapeutic dose, without the need of repeated administrations.

## Materials and Methods

### Materials

#### Chemical Materials

ACV was kindly offered by Labesfal Genéricos® and ω3 fatty acids were purchased from MyProtein®(Portugal). PCL (MW = 80 kDa) was obtained from Sigma-Aldrich Química, S.L. (Sintra, Portugal). All reagents and solvents were of highest purity available and spectroscopic grade and were purchased from Sigma-Aldrich Química, S.L. (Sintra, Portugal), unless otherwise indicated.

#### Cell Materials

Cell materials the primary human epidermal keratinocyte HaCaT cell line was obtained from ATCC (Manassas, VA, USA). Dulbecco's Modified Eagle's Medium was acquired from Lonza (Verviers, Belgium) and supplemented with 10% (v/v) fetal bovine serum (Merck Millipore, Burlington, MA, USA); 100 U/mL penicillin (Merck Millipore) and 100 μg/mL streptomycin (Merck Millipore). 3-(4,5-dimethyl-2-thiazolyl)-2,5-diphenyltetrazolium bromide (MTT) used in the cytotoxicity assays was acquired from Sigma-Aldrich Química, S.L. (Sintra, Portugal). Cells were maintained and used throughout experiments in complete medium and under routine cell culture conditions (37°C, 5% CO_2_, and 95% RH).

### Preparation of Electrospun Polymeric Fibrous Matrices

To prepare the PCL electrospinnable solution, 0.6 g of PCL was dissolved in 5 mL of solvent mixture composed of methanol and chloroform (3/2) (v/v). To prepare the electrospinnable solution for ACV-loaded PCL fibers, ACV was used at loading percentages of 8 and 17% of the initial polymer weight (0.6 g) and dissolved in the same solvent mixture. Polymeric solution used for preparation of electrospun fibrous matrices with ACV and ω3 was achieved at loading percentages of 17 and 33%, respectively, of the initial polymer weight (0.6 g) and dissolved in the same solvent mixture. The polymeric solutions without or with ACV or ACV and ω3 were then stirred thoroughly to form a homogenous spinning solution.

Before the production of the electrospun fibrous matrices the processing parameters, such as the intensity of the applied electric field, distance between injection and collector, flow rate, and internal diameter of the needle were optimized. The optimization of these parameters is herein briefly presented, but for a more extensive explanation about the optimization parameters and their influence on final fibrous matrices, please refer to good reports on the subject (Huang et al., 2003; Beachley and Wen, 2009; Haider et al., 2018). The intensity of the applied electric field is an important factor in the optimization of the electrospinning process, since it must be higher than the surface tension so that a jet formation occurs from Taylor's cone (Huang et al., 2003). Therefore, an optimal voltage value was found to allow the formation of the polymeric jet and prevent dripping of the polymeric solution. The voltage values applied were also combined with tests of the optimal distance between the needle and collector, as an increasing distance favored solvent evaporation but required higher voltage. The flow rate of ejection of the polymeric solution controls the formation of the drop from which the Taylor cone is formed, and it is related to syringe emptying speed. So, the bigger the flow rate, the larger the drop formed, and thus a larger dispersion of charges and instability of the polymeric structure occurs, boosting aggregate formation. On the other hand, slower flow rates favored greater solvent evaporation but if too slow there was a needle occlusion due to polymeric solidification at the needle end. As such, an optimal flow rate was adjusted to allow continuous emptying of the syringe and avoid instability of the polymeric structure or needle clotting. The inside diameter of the needle also influences the size of the drop formed and was chosen to enable the formation of a continuous jet. After these considerations regarding the optimization procedure, the electrospinning parameters were set as follows: the applied voltage of 15 kV, the collection distance of 12 cm, and the solution flow rate of 0.6 mL/h controlled by a syringe pump (SP100Z, WPI—World Precision Instruments, United Kingdom) that ejected the polymeric solution placed into a 5-mL plastic syringe with a blunt-ended needle with 0.6 mm inner diameter.

All the electrospinning parameters were kept constant and all the experiments were conducted at 18–22°C. The collected fibrous mats were dried at least 72 h at 37–40°C to fully remove the solvent residue, before the experiments.

### Characterization of Polymeric Fibrous Matrices

Several methods were used to characterize the produced fibrous matrices. The surface topography, morphology, and diameters distribution of the electrospun fibers were evaluated by scanning electron microscopy (SEM). SEM images were obtained on a Phenom ProX equipment (Paralab, Portugal) using the backscattered electrons detector (BSD). An image analysis software, Digimizer® (version 4.6.1, MedCalc Software), was used for manual and accurate measurement of the fiber's diameters (calculated from the measurements of at least 1,000 fibers). SEM images were also used to measure the thickness of the fiber mats, which enables to estimate the fibrous matrix porosity (p), using equation (1):

(1) $$\begin{array}{r} p\;=100\left(1-\;\left(\frac{\rho_{fibers}}{\rho_{PCL}}\right)\right) \end{array}$$

where ρ_*PCL*_ is the density of PCL (1.145 g/cm^3^ as indicated by the supplier) and ρ_*fibers*_ is the effective density of the fiber mats, obtained by dividing the mass of a sample by its volume.

Water contact angles on the surface of the electrospun fiber mats were measured on a Drop Shape Analysis System DSA100 equipped with a CCD camera.

The mechanical response of the polymeric fibrous matrices was studied by carrying out stress-strain tests using an AGS-X Shimadzu 500N equipment with a 10 N load cell and a deformation speed of 10 mm/min at room temperature.

To assess the chemical composition of the fibrous matrices with and without ACV and ω3, and confirm the incorporation of ACV, attenuated total reflectance Fourier transform infrared spectroscopy (ATR-FTIR) was used. ATR-FTIR was measured in a Perkin-Elmer Spectrum Two Spectrometer equipped with an attenuated total reflection unit using a diamond crystal.

Structural characterization of the fibrous mat was achieved by X–ray diffraction (XRD) using a Philips Diffractometer, model PW1710.

Thermal analysis was performed by differential scanning calorimetry (DSC) in a DSC 3+ Mettler Toledo. Measurements were carried out under nitrogen atmosphere with a flow rate of 20 mL/min.

### Evaluation of the Potential Use of the Polymeric Fibrous Matrices for Topical Applications

In order to evaluate the potential use of the developed polymeric fibrous matrices for topical applications, four *in vitro* studies were performed: drug release assays under biological mimetic conditions, skin permeation tests, evaluation of occlusive efficiency and cell viability experiments using the MTT reduction assay.

#### Evaluation of the Release Profile of Bioactive Compounds From the Polymeric Fibrous Matrices

For ACV release studies, a fiber mat sample was cut, weighted, and put into a dialysis bag with a cut-off of 3.5 kDa and exposed to the release medium. The release medium was a micellar dispersion (sodium lauryl sulfate 16 mM, above the critical micellar concentration described for this surfactant Mukerjee and Mysels, 1971) at pH of 5.5 (prepared from a basic solution of sodium phosphate decahydrate 0.1 M and an acid solution composed by boric acid 0.02 M and citric acid 0.05 M Perrin and Dempsey, 1974) to mimic not only the pH value of the skin (Fluhr and Elias, 2002), but also the membranous/aqueous environment encountered in the interlamellar regions of the *Stratum corneum* that constitutes one of the most important barriers for drug permeation (Lopes et al., 2018). Taking in consideration that viral infections promote an inflammatory process with consequent increase in the temperature of the skin (Chanmugam et al., 2017; Lopes et al., 2018) studies were carried out at 37°C under constant stirring of 100 rpm (IKA, RO10, Reagente5, Portugal). Aliquots of 1 mL of the released medium were taken at predetermined time points, while an equal amount of fresh medium was added back to assure sink conditions. All the ACV release experiments were performed at least in triplicate and the results were expressed as means values ± SD. The amount of ACV released at each time point was quantified at λ = 252 nm by validated UV/Vis spectrophotometry method accordingly to International Conference on Harmonization guidelines in UV-VIS spectroscopy (ICH, 2005) in a Shimadzu UV-2501 PC spectrophotometer. The cumulative ACV released was calculated and reported as a percentage of the theoretical maximum of drug content and represented as a function of time. The drug release profile of a commercial formulation containing ACV (5%), Zovirax™ cream (GlaxoSmithKline, Algés, Portugal) was also determined. The *in vitro* release profile of ACV from the fibers and from commercial formulation was fit with different mathematical models to evaluate the release kinetics and elucidate the mechanisms that control drug release from the dosage forms (Costa and Lobo, 2001). Models used were first-order, Higuchi, Korsmeyer-Peppas and Gallagher-Corrigan (Gallagher and Corrigan, 2000; Costa and Lobo, 2001). The model with highest value of adjusted coefficient of determination (*R*^2^ adjusted) (Costa and Lobo, 2001) was selected as the model which best fits to the release profile of ACV. The GraphPad Prism^®^ 5.03 software was used as the curve fitting system to calculate the *R*^2^ and model parameters.

Given the high lipophilicity of ω3, its release is only possible upon fiber close contact with skin surface and it is not possible to simulate in an aqueous release media. The release of ω3 was thus achieved in an organic solvent following the procedure used to evaluate the *in vitro* release of fish oils from microcapsules (Augustin et al., 2003). The use of *n*-hexane, an organic solvent that mimics the hydrophobic part of the cell membrane, being one of the widely used lipid-like organic phase in partition coefficient studies (Ruelle, 2000) provided a chemical environment that is similar to the non-polar media encountered in high lipid content of skin where ω3 will solubilize providing a proof that the fish oils fatty acids were successfully incorporated in the polymeric matrix of the fibers. The amount of ω3 released at each time point was quantified at λ = 269 nm by a similar procedure to the one described for ACV.

#### Evaluation of Occlusive Factor and Permeation Properties of the Polymeric Fibrous Matrices

To evaluate how ACV permeates the skin, a permeation assay using Franz diffusion cells and pig skin was used (Allen et al., 1989; OECD/OCDE, 2004). Porcine tissues were immediately collected after pigs were sacrificed from a local slaughterhouse and adipose tissues and connective tissues were removed using surgical scissors. The permeation area was 2.54 cm^2^, and the volume of receptor media (micellar dispersion at pH 5.5) was 5 mL stirred at 600 rpm and temperature of 37°C. Franz diffusion cell was equilibrated for 30 min after mounting the pig skin. PCL fibers loaded with ACV were placed in donor container over the pig skin and the lower part of the skin contacted with the receiving medium (micellar dispersion at pH 5.5). The skin permeation of ACV from Zovirax™ cream was also tested. Samples (0.4 mL) were obtained at different time intervals (1, 2, 4, 6, 8, 12, 24, and 36 h) from the receptor part and ACV quantified as described above by validated UV/Vis spectrophotometry method (ICH, 2005). Permeation parameters were interpreted from the cumulative amount of released drug per unit skin area (Q_R_/A) vs. time (t) plot. The gradient and x intercept of the linear portion of the plot yield steady-state flux (J_ss_) and lag time (t_L_), respectively. Steady-state flux of drug, J_ss_ (μg/cm^2^h) was calculated by equation (2), where t (h) is permeation time, A (cm^2^) is permeation area, and *Q*_R_ (μg) is permeated amount of ACV:

(2) $$\begin{array}{r} J_{SS}\;=\frac{Q_{R}}{At} \end{array}$$

The drug permeability K_p_ (cm/h) was calculated by the following equation (3), where C_d_ is the initial concentration in the donor chamber (μg/cm^3^):

(3) $$\begin{array}{r} K_{P}\;=\frac{J_{SS}}{C_{d}} \end{array}$$

The water permeability is a basic physical property of wound dressings that may influence the wound healing process (Hinman and Maibach, 1963; Atiyeh et al., 2010) and for that reason the occlusive effect resultant from skin coating with the fibrous scaffold should be investigated. The occlusive effect results from the prevention of the outflow of water, maintaining its hydration. The *in vitro* occlusion test was adapted from the literature (Souto et al., 2004). Briefly, test glass containers were filled with an accurate mass of water. The top of each container was covered with either a microfiber cellulose filter (cellulose filter, 70 mm, Whatman, Sigma-Aldrich, Química, S.L., Sintra, Portugal; cutoff size: 8–12 μm) (control samples) or by placing fibers mat (with the same area of the filter) upon the filter. Zovirax™ cream formulation was also tested by uniformly spreading on the filter a precise mass of cream. Each system was sealed and placed at 37°C being accurately weighed at defined time points to evaluate the water loss due to evaporation at each time (water flux through the filter paper). The occlusive factor (F) was calculated using equation (4):

(4) $$F\;=\left(\frac{\Phi_{\left(\text{w}/\text{o}\right)}-\;\Phi_{\left(\text{w}\right)}}{\Phi_{\left(\text{w}/\text{o}\right)}}\right)$$

Where **Φ** is the water flux (percent water loss) through the uncovered filter (w/o) or through the filter when covered (w) by the system under evaluation (polymeric fibrous scaffolds or acyclovir-cream commercial formulation) (Souto et al., 2004; Etxabide et al., 2017).

Water vapor transmission rate (WVTR) was calculated with equation (5), where t represents the 24 h time point, A is the sample testing area (m^2^) and W_i_ and W_f_ are the initial and the final weight (on 24 h time) of the system, respectively (Etxabide et al., 2017):

(5) $$\begin{array}{r} \text{WVT}\text{R}\;=\frac{{\text{w}}_{\text{i}}-\;{\text{w}}_{\text{f}}}{\text{t}\text{A}} \end{array}$$

The assays were performed in triplicate and the results were expressed as means values ± SD.

#### Evaluation of the Cytotoxicity of the Polymeric Fibrous Matrices

The MTT reduction assay was used to evaluate the *in vitro* cytotoxicity of PCL polymeric fibers, either unloaded or co-incorporating ACV and ω3. The methodology was adapted from ISO guidance on the biological evaluation of medical devices (ISO 10993-5, 2009). Extracts from fibers were obtained by incubation with cell culture medium (100 mg of fiber per mL of medium) at 37°C for 24 h, under orbital shaking (100 rpm). The assay was performed by testing the toxicity of extracts to epidermal keratinocytes (HaCaT). Cells (1 × 10^4^ cells/well) were incubated in 96-well plates for 24 h before plain medium was replaced by the extracts and allowed to incubate for an additional 24 h. A negative control (mock extract) and a positive control (1% Triton X-100 extract) were also included. After incubation, the extracts were removed and, after washing the cells with PBS, a solution of MTT in medium (0.5 mg/mL) was added. The cells were incubated for 4 h in the dark in order to incorporate MTT and metabolize it into formazan crystals. These last were solubilized by DMSO and quantified spectrophotometrically at a wavelength of 570 nm (readings at 630 nm were used for background deduction). The results were expressed as percentage cell viability from negative control.

## Results

### Characterization of Polymeric Fibrous Matrices

#### Morphology, Fiber Average Diameter, Porosity, and Surface Properties

**Figure 2:**
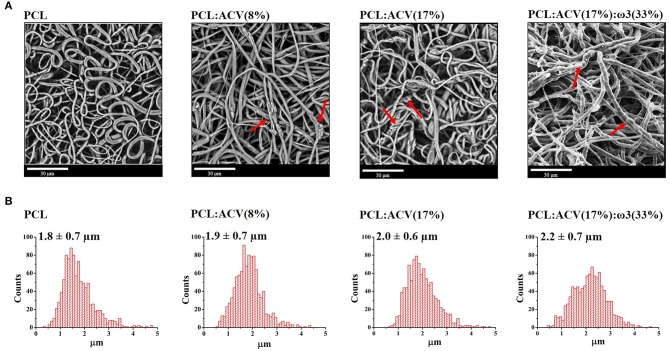
Morphologic characterization of polymeric fibrous matrices: **(A)** SEM images of fibrous matrices for unloaded and loaded fibers with different bioactive composition. **(B)** Histogram of the diameters distribution of fibers for the different fibrous matrices analyzed (*n* = 1,000). Bioactives concentration is expressed in wt% relatively to the polymer. Red arrows indicate points of rough and/or wrinkled surfaces indicative of morphological alterations.

SEM images of polymeric fibrous matrices, unloaded (PCL), loaded with ACV [PCL:ACV(8%) and PCL:ACV(17%)] and loaded with ACV and ω3 [PCL:ACV(17%):ω3(33%)], are presented in Figure 2.

The PCL fibrous matrix is constituted by randomly oriented fibers with a smooth surface, a cylindrical morphology (Figure 2A, PCL) and an average diameter of 1.8 ± 0.7 μm (Figure 2B, PCL).

Loaded polymeric fibrous matrices maintained the cylindrical morphology of the PCL fibers although presenting rough and/or wrinkled surfaces in some places (red arrows in Figure 2A) which become more apparent with higher concentration of ACV [red arrows in Figure 2A, PCL:ACV(17%)] or with the loading of both ACV and ω3 [red arrows in Figure 2A, PCL:ACV(17%):ω3(33%)]. Furthermore, upon loading of increasing amounts of ACV or co-loading of ACV and ω3, the average diameter of the fibers slightly increases from 1.8 ± 0.7 μm in unloaded fibers, to 2.2 ± 0.7 μm in fibers loaded with both active compounds (Figure 2B). This morphological alteration and increase in fiber diameter have been observed by other authors upon drug loading into fibers (Shen et al., 2011; Ardeshirzadeh et al., 2015; Yu et al., 2015) and can be attributed to changes in the viscosity and electrical characteristics of the polymeric solution after co-solubilization of the active compounds. However, the changes were not so relevant to require an optimization of the electrospinning process and the establishment of new electrospinning parameters when producing fibers loaded with the bioactives.

Since a moderate effect in the morphology and diameter of the PCL fibers was observed upon loading of the bioactives, we analyzed if the incorporation of the bioactives had consequences in changing the porosity and the surface properties of the polymeric matrix, as these are two important properties for wound dressing purposes and thus important for herpes lesions healing. The porosity of the fiber mats was estimated from the apparent density by weighing and measuring the area and thickness of representative samples of the unloaded and loaded polymeric matrices. In comparison with unloaded PCL mats, the loaded ones were thinner (Supplementary Figure 1A) and presented a decrease in the estimated porosity (74% instead of 93%, as shown in Table 1) though keeping a high porosity. The static water contact angles (Supplementary Figure 1B) were however similar and over 90° for both unloaded and loaded polymeric matrices (Table 1) and were also in agreement with the reported values for this polymer in a related morphology (Ranjbar-Mohammadi and Bahrami, 2015). This indicates that the addition of the bioactives does not significantly change the hydrophobicity of the PCL fibrous mats. It also suggests that both bioactives must be incorporated in the PCL matrix core. Indeed, the value of ACV octanol/water partition coefficient (log P = −1.59 Garré et al., 2007) shows that this drug is quite hydrophilic. So, it would be expected that, if the drug was located on the surface of the fibers, it would lower the value of the contact angle with the water. The same rational applies to ω3, which is highly lipophilic (logP = 6.10, calculated by chemicalize tool by ChemAxon®), and would increase the contact angle of PCL if located at the surface of the fibers.

**Table 1:** Characterization of PCL fibrous matrices without and with bioactive compounds (17 wt% of ACV and 33 wt% of ω3).

**Polymeric fibrous matrices**	**Fibers average diameter (μm)**	**Thickness of the fibrous mat (μm)**	**Porosity (%)**	**Static water contact angle (**°**)**	**Maximum tensile strength at break (MPa)**	**Elongation at break (%)**	**Young's modulus (MPa)**	**T_**f**_ (^**°**^C)**	**ΔH_**f**_ (J/g)**
PCL	1.8 ± 0.7	364	93	129.7 ± 2.3	5.1 ± 0.9	96.8 ± 9.3	5.2 ± 0.4	56.7	56.3
PCL:ACV:ω3	2.2 ± 0.7	315	74	129.2 ± 1.6	4.1 ± 0.1	107.3 ± 9.9	3.8 ± 0.3	57.1	37.2

Table 1 summarizes the main characteristics of the polymeric fibrous matrices unloaded and loaded with ACV and ω3.

#### Mechanical Properties

Polymeric fibrous matrices integrating bandages for herpes lesions healing should be resistant to manipulation and thus their mechanical resistance was tested by stress-strain assays.

Supplementary Figure 2 shows typical tensile strain-stress curves of PCL fibrous matrices unloaded (PCL) or loaded with the bioactive compounds [PCL:ACV(17%):ω3(33%)]. The mechanical properties of these fibrous matrices are summarized in Table 1. The characteristic strain-stress curves are typical of a thermoplastic material, where it is possible to identify an elastic behavior for lower applied stresses and a plastic behavior for higher stresses, before the breaking of the sample. Overall, the addition of the bioactive compounds does not significantly modify the mechanical characteristics of the fibers, as confirmed by the values of maximum tensile strength and elongation at break (Table 1). Indeed, in both unloaded and loaded fibers, there is an elastic deformation of about 10% upon applied tensile stress of 2–2.5 MPa (Supplementary Figure 2). Moreover, up to the rupture, both unloaded and loaded fibers elongated about 100% with applied stresses in the order of 4–5 MPa (Table 1). There was however a slight decrease in Young's modulus in PCL:ACV(17%):ω3(33%) fibers, indicating a reduction of tensile strength upon bioactives incorporation in the fibers. All in all, in agreement with other reported studies (Ferreira et al., 2014; Ranjbar-Mohammadi and Bahrami, 2015) PCL fibrous matrix revealed a high mechanical resistance and the inclusion of the bioactives did not significantly affect the mechanical resistance of PCL fibers.

#### Chemical, Structural, and Thermodynamic Properties

**Figure 3:**
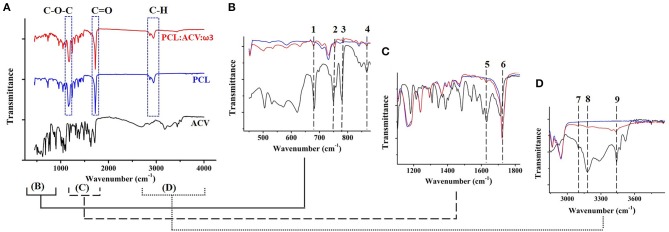
ATR-FTIR spectra of ACV raw material (black spectra), PCL fibers unloaded (blue spectra), and PCL loaded fibers with the bioactives (PCL:ACV:ω3, red spectra). **(A)** ATR-FTIR spectra from 450 to 4,000 cm^−1^. **(B)** ATR-FTIR spectra from 450 to 880 cm^−1^. **(C)** ATR-FTIR spectra from 1,100 to 1,430 cm^−1^ **(D)** ATR-FTIR spectra from 2,840 to 3,880 cm^−1^. Dashed rectangles represent the characteristic PCL fingerprints. The numbers and the dashed lines represent the different fingerprints of ACV identified in PCL:ACV:ω3 fibrous matrices.

The chemical structure of PCL fibers before and after the loading of the bioactives was characterized using ATR-FTIR. The ATR-FTIR spectra of ACV raw material (ACV, black spectra) and PCL fibers unloaded (PCL, blue spectra) or loaded with the bioactives (PCL:ACV:ω3, red spectra) are shown in Figure 3.

In agreement with the reported literature (Elzein et al., 2004; Borjigin et al., 2013), PCL fibrous matrices, unloaded or loaded with the bioactives, exhibit the three main characteristic bands attributable to the polymer (Figure 3A, bands marked with a dashed square): one, located at 1,166 cm^−1^, attributed to symmetric C–O–C bond stretching of the repeated monomers within the PCL polymer, another, very strong, located at 1,723 cm^−1^ and resulting from carbonyl (C=O) bond stretching and a broad band at 2,940–2,860 cm^−1^ attributed to C–H stretching.

Loading of ω3 could not be confirmed by ATR-FTIR, as it was not possible to identify any bands specifically attributed to this fatty acid in the IR spectrum of PCL fibers loaded with the bioactives (Figure 3A). Indeed, the chemical structure of ω3 resembles PCL chemical structure and has common groups with the polymer (e.g., C=O and C-H bonds) (Figure 1). Therefore, the ATR-FTIR vibration bands more characteristic of ω3 (Bekhit et al., 2014) are analogous to the ones found in PCL (Supplementary Figure 3).

The successful loading of ACV in PCL was qualitatively confirmed by identification of vibration modes, characteristic of the drug, in the ATR-FTIR spectrum of PCL fibers loaded with the bioactives, being these bands absent in the ATR-FTIR spectrum of unloaded PCL fibers. For a clearer comparison, ATR-FTIR spectra of Figure 3A was divided in three IR regions, presented as insets in Figures 3B–D. In the fingerprint IR region (Figure 3B) it is difficult to assign bands to specific vibrations. However, in this region it is possible to observe four bands (marked as 1, 2, 3, and 4 in Figure 3B) that are present in both ACV and drug-loaded PCL fibers spectra, being absent in the spectrum of unloaded PCL fibers. Another distinctive region is the correspondent to Figure 3C where the band at 1,634 cm^−1^ (band 5 in Figure 3C) is assigned to the bending vibration of ACV primary amine. In agreement with the literature (Sawdon and Peng, 2014), this band appears in the spectrum of ACV and is also detected in the spectrum of loaded PCL fibers, but it is not visible in the spectrum of unloaded PCL fibers, confirming the successful incorporation of this drug. The C=O stretch band of ACV that, in agreement to the literature (Chen and Yu, 2010; Yu et al., 2010), appears at 1,698 cm^−1^ (band 6 in Figure 3C), does not appear in loaded in PCL fibers, probably by being overlapped by the band of the carbonyl group bond stretching of the polymer. Finally, the bands of ACV spectrum in the IR region of 3,200 to 3,600 cm^−1^ (Figure 3D, bands 7, 8 and 9) can be ascribed for N–H stretching vibration of primary and secondary amines or to O–H stretching vibrations which appear in the same region (Mukherjee et al., 2007). These bands do not appear in the IR spectrum of PCL unloaded fibers but are present at IR spectrum of PCL loaded fibers, thus constituting another proof of successful ACV loading in the fibers.

**Figure 4:**
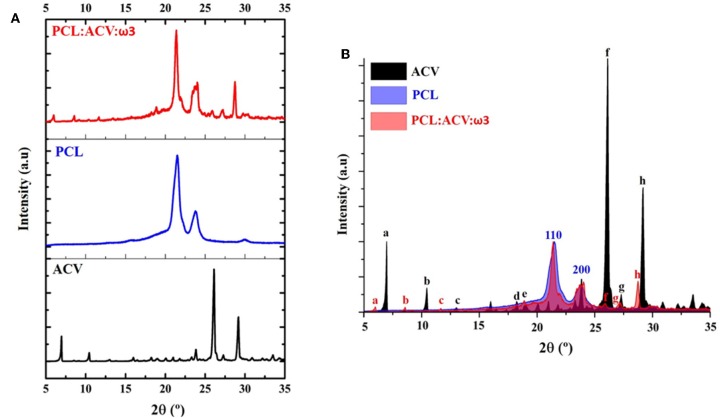
**(A)** XRD diffractograms of ACV raw material (black) and PCL fibers unloaded (blue) or loaded with the bioactives (PCL:ACV:ω3, red). **(B)** Superimposition of XRD diffractograms. Blue numbers (110, 200) represent the position of diffraction plans of PCL in unloaded fibers and are also present in loaded fibers with bioactive compounds. Black letters assign the position of diffraction peaks of ACV raw material which are identified and assigned with red letters in PCL loaded fibers (PCL:ACV:ω3).

The crystalline structure of PCL fibers before and after the loading of the bioactives was characterized using XRD. The XRD diffractograms of ACV (ACV, black spectra) and PCL fibers unloaded (PCL, blue spectra) or loaded with the bioactives (PCL:ACV:ω3, red spectra) are shown in Figure 4.

The diffraction pattern of ACV raw material (Figure 4A, black diffractogram) presented several peaks, some of which situated at a 2θ angle of 6.9°, 10.4°, 23.9°, 26.1°, and 29.2° coherent with a polymorphic form V of this drug (Lutker et al., 2011). In agreement with the literature (Wang et al., 2013; Ferreira et al., 2014) the pattern of unloaded PCL fibers (Figure 4A, blue diffractogram) presented two characteristic intense peaks situated at a 2θ angle of 21.5° and 23.8° due to diffraction by the (110) and (200) planes, respectively, indicating a crystalline orthorhombic structure of the polymer in the fibers. Diffraction by the (210) and (211) planes result in two peaks that appear convoluted at one single 2θ angle of 30°. These three peaks are also observed in the diffractogram of PCL loaded fibers (Figure 4A, red diffractogram) suggesting that the inclusion of the bioactives did not affected the crystallinity of the polymer to a great extent.

For a better comparison between the diffractograms, Figure 4B presents their superimposition. In Figure 4B it is noticeable that apart from the diffraction peaks characteristic of the polymer (assigned as 110 and 200), the XRD pattern of PCL loaded fibers shows numerous additional peaks (assigned with red letters “a” to “h”) that closely correspond to those of ACV raw material (assigned with black letters “a” to “h”), although being shifted to lower angles. The shift in peaks position may be attributed to the stretch stress to which the ACV is subjected during the manufacturing process of the fibers. The peaks observed in PCL loaded fibers are thus likely ascribed to ACV diffraction, indicating the successful drug loading. Additionally, the stretch stress may have caused in the drug crystals a certain texture, i.e., the existence of preferential growth orientations of ACV crystals when loaded into the polymeric fibers. This can be concluded by changes in relative intensities of ACV diffraction peaks when ACV is included in the polymeric fibers.

After the chemical and structural characterization, DSC was carried to inform about changes in energy transitions in the PCL matrices upon bioactives incorporation. DSC analysis was performed on unloaded and loaded PCL fibrous matrices (Supplementary Figure 4) and the corresponding thermodynamic parameters (melting temperature of the polymer, T_f_ and correspondent enthalpy change, Δ*H*) are presented in Table 1. Unloaded PCL fibers presented the characteristic melting peak of the polymer, at around 57°C in agreement with other reports (Rusu et al., 2006). This temperature is maintained after inclusion of the bioactives in the polymeric matrix. However, a small decrease in the Δ*H* value, by about 19 J/g, indicates that the incorporation of ACV and ω3 in the fibers, although not compromising their structural integrity, led to a small decrease in its crystallinity. This corroborates the results obtained in mechanical tests that point for a small reduction of the Young's modulus upon incorporation of the bioactives in the polymeric fibrous matrix. The individual effect of each bioactive in the thermal transition of PCL fibers (Supplementary Figure 4 and Supplementary Table 1) is similar to the effect observed when both actives were loaded in the fibrous matrix (Table 1). Moreover, the decrease of Δ*H* value observed for PCL fibers only loaded with ω3 suggests its successful loading into the polymeric matrix.

From the combined analysis of the results obtained by the three techniques, we may conclude that although a crystallinity reduction effect is observed in the polymeric matrix upon the inclusion of the bioactives, this effect is small and does not compromise the mechanical resistance and structural integrity of the fiber mat. Ultimately this also suggests that PCL fibrous matrices are adequate for the co-incorporation of the two selected bioactives.

### Evaluation of the Potential Use of the Polymeric Fibrous Matrices for Topical Applications

To ensure that the developed polymeric matrices are adequate bandages for herpes lesions healing, it is required that several parameters, considered as important predictors of the use of the developed system, can be evaluated. These include the *in vitro* evaluation of: (i) the occlusive factor responsible for maintaining the hydration required for proper healing; (ii) the release profile of the bioactives, to guarantee an adequate dose for achieving a therapeutic effect (iii) skin permeation tests to assess the transdermal drug delivery; and (iv) the cytotoxicity to test the potential safety of the topical system.

#### Occlusive Factor of the Polymeric Fibrous Matrices

A dressing is said to be occlusive if a moisty wound surface is maintained when the dressing is in place. Occlusive or semi-occlusive dressings prevent wound desiccation by water retention on skin surface, thus reducing evaporation losses. It is well-documented in the literature that occlusive properties in dressings accelerates the healing of partial-thickness wounds, such as the resultant from HSV infections (Patel et al., 2007).

The *in vitro* evaluation of the occlusion potential of PCL fibrous matrix revealed that it is able to hinder water evaporation in comparison to the control system used (microfiber cellulose filter). The occlusive factor of PCL fibrous matrix, calculated by equation (4), was about 12% at 24 h and was maintained at about 10% over 108 h of evaluation (Supplementary Figure 5). The value of the WVTR, calculated according to equation (5), was 881 ± 91 g/m^2^day. PCL fibrous matrix also revealed significantly higher prevention of water loss than that of the commercial ACV formulation (Zovirax® cream) for which the water evaporation was higher than that of the control system, due to the evaporation of water from the formulation, displaying non-occlusive capacity.

#### Bioactives Release Profile From the Polymeric Fibrous Matrices

**Figure 5:**
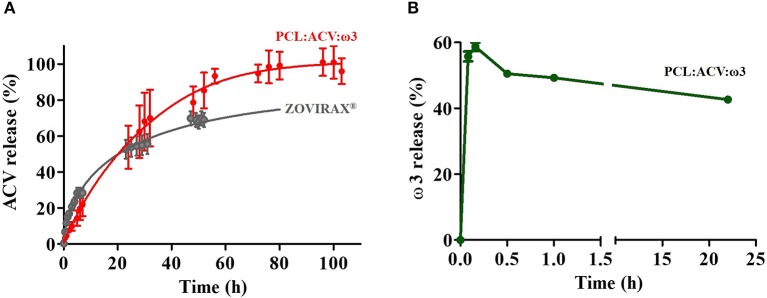
**(A)** Release profile of ACV from PCL loaded fibers (PCL:ACV:ω3) (red) and from commercial cream formulation (Zovirax) (gray) in micellar medium (37°C at pH 5.5). **(B)** Release profile of ω3 from PCL loaded fibers (PCL:ACV:ω3) (green) in hexane. The bars represent the standard deviation (*n* = 3).

For therapeutic applications, the incorporated ACV and ω3 loaded in the PCL fibrous matrix must be released to exert their biological function. Therefore, we evaluated the release profiles of ACV (Figure 5A) and ω3 (Figure 5B) from the PCL fibrous matrix. As a control, the release of ACV from Zovirax® cream was also investigated.

From analysis of Figure 5A it is possible to conclude that PCL fibrous matrices provide sustained ACV release and the total drug content is reached at 96 h. The profile of ACV from the commercial formulation shows faster drug release in the first hours of the assay. Indeed, in the first 3 h, ACV released from commercial formulation is about 2 folds more than ACV released from the polymeric system. However, after 23 h of assay, higher release of ACV from the fibrous system is observed. To further assess the release mechanism of ACV loaded either in the fibrous matrix or in the commercial formulation, both release profiles were adjusted to representative kinetic models, namely, first-order, Korsmeyer-Peppas, Higuchi and Gallagher-Corrigan (Gallagher and Corrigan, 2000; Costa and Lobo, 2001). The evaluated kinetic release parameters are listed in Supplementary Tables 2–5. Results of these analyses show that the best fits to the experimental data, with *R*^2^ values of >0.99 were obtained with first-order for the PCL fibrous matrices and with the Korsmeyer-Peppas model for the commercial formulation, which are the fittings presented in Figure 5A. The Gallagher-Corrigan model also provided a good fit for both systems and can be used for a more accurate comparation of ACV release profile (Supplementary Table 5). This model is adequate to describe the drug being released in a biphasic manner, combining contributions from a first-order initial release phase that is faster and is followed by a slower controlled release phase (Gallagher and Corrigan, 2000). As previously mentioned, in an initial stage, ACV is released in a faster way from the commercial formulation. This is reflected in the higher value of the release rate constant (k_1_) observed in the commercial formulation (k_1zovirax_ = 0.333 ± 0.077 h^−1^ and k_1PCLfibers_ = 0.0391 ± 0.0103 h^−1^). The reason for this difference is obvious, since in the commercial formulation ACV is freely dispersed in an oil/water emulsion and thus more readily available to diffuse to the release media. Conversely, ACV in the PCL fibrous matrices is in the polymeric core of the fibers and thus its diffusion to the release media is slower. In a second stage, the release rate constants of ACV from both systems are higher and identical within the error (k_2zovirax_ = 0.118±0.019 h^−1^ and k_2PCLfibers_ = 0.0767±0.0231 h^−1^) indicating that a slower release phase is occurring in both systems. In this phase the drug, deeply loaded in the porous polymeric matrix, is released due to the swelling in contact with the aqueous release medium and/or the slow erosion of the polymer matrix (Gallagher and Corrigan, 2000). The ACV release is also more complete from the fibrous matrix and about 80% was released by 36 h while the commercial formulation released only 27% at 23 h (see F_b_ and T_max_ values in Supplementary Table 5). One possible explanation for this distinct release behavior appears to be the large surface area of the fibers allowing a wider dispersion of drug on the fiber which facilitates a more complete diffusion of the drug into the dissolution medium.

The release of ω3 (Figure 5B) reached about 60% within 10 min of contact of the fiber with the solvent decreasing afterwards. In release assays a steady state condition is established relatively quickly for low molecular weight drugs, so the solution drug concentrations increase in the release medium until an equilibrium is reached. This might not be the case for higher molecular weight compounds such as ω3, which could explain the atypical release profile. PCL is described as a very lipophilic polymer (logP = 4.03) (Magenau et al., 2015) capable of incorporating poorly water soluble (highly lipophilic) drugs or bioactives such as ω3 (logP = 6.10 calculated with chemicalize tool from Chemaxon® software). The affinity of the ω3 for PCL and for the release solvent should be very similar, since this solvent and this polymer have very close logP values (logP (hexane) = 3.90) (Hansch et al., 1996). This justifies the initial fast release of ω3 by contact with the solvent (about 60% in the first 10 min). The subsequent decrease (to about 40% at the end of the 22 h assay) may probably be attributed to an equilibrium establishment with a redistribution of the bioactive compound between the release media and the polymer matrix with which it has high affinity. We should also highlight that, often by the impossibility of release assays replicate the lipophilic content that exists *in vivo*, authors use organic solvents (Augustin et al., 2003), that are not mimetic of any *in vivo* solvent, but that have similar polarity/lipophilicity allowing to predict that the lipophilic compounds will be released under appropriate conditions. Therefore, the solvent here used was chosen because it does not solubilize PCL (Bordes et al., 2010) and it provides a highly nonpolar lipophilic media. All in all, this assay indicates that ω3 was loaded in the fibers and could be released from the fiber matrix upon the presence of highly non-polar lipophilic media such as the one found in the rich lipid matrix that lies between the corneocytes of the *Stratum Corneum* (Lopes et al., 2018).

#### Skin Permeation of ACV Loaded Polymeric Fibrous Matrices

Although it is well-known that the herpes virus affects keratinocytes, what is less well-studied is that, pathology is extended to the dermis (Patel et al., 2007) and thus it is also important to assess the drug permeation capacity.

**Figure 6:**
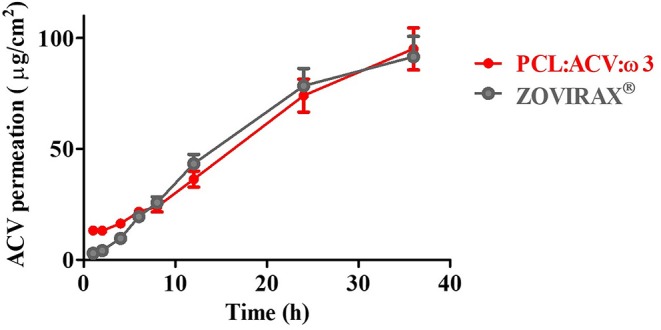
*In vitro* ACV permeation of pig skin from PCL loaded fiber (PCL:ACV:ω3) (red) or from commercial cream formulation (Zovirax) (gray). Receptor compartment of Franz cells was filled with micellar medium (37°C at pH 5.5).

Comparison of the *in vitro* permeation profiles for ACV from PCL fibrous matrices and from Zovirax® are shown in Figure 6.

From the permeation profiles obtained it was possible to calculate different permeation parameters that allow to quantitatively compare the two systems (Table 2).

**Table 2:** Skin permeation parameters of ACV obtained for PCL fibrous matrices and for the commercial formulation Zovirax®.

**Formulation**	**Steady-state flux of drug, Jss (μg/cm^**2**^h)**	**Lag time, t_**L**_(h)**	**Drug permeability, Kp (cm/h)**
PCL:ACV:ω3	2.120 ± 0.198	9.131 ± 1.316	0.00928 ± 0.000867
Zovirax®	3.088 ± 0.166	0.000 ± 1.397	0.00794 ± 0.000427

In agreement with the drug release results, the permeation of ACV across skin was faster (J_ss_ = 3.088 ± 0.166 μg/cm^2^h) for the commercial formulation which also permitted an immediate release of the drug (t_L_ = 0). On the other hand, PCL fibrous matrix retarded the flux rate of drug across the skin (J_ss_ = 2.120 ± 0.198 μg/cm^2^h and t_L_ = 9 h) but this system improved ACV permeation (Kp = 0.00928 ± 0.000867 cm/h) in comparison with the commercial formulation (Kp = 0.00794 ± 0.000427 cm/h). Relatively to the reported for free ACV drug (not included in a formulation) (Lopes et al., 2018) both the commercial formulation and PCL fibrous matrices were able to improve the drug permeation up to 18 or 21 times, respectively.

#### Cytotoxicity of the Polymeric Fibrous Matrices

**Figure 7:**
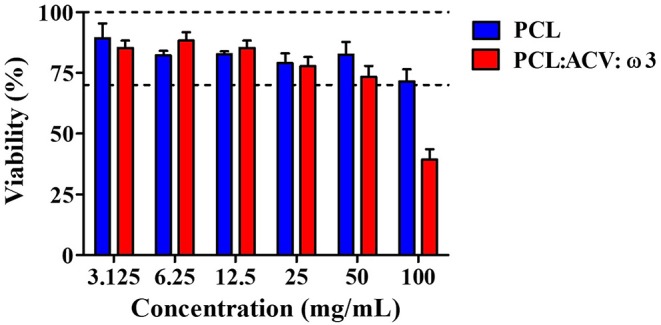
Cellular viability determinated by MTT assay for HaCaT cell lines after 24 h of exposure to extracts of unloaded PCL fibers (blue columns) and PCL loaded fibrous matrices with ACV and ω3 (red columns). The columns represent medium values and the bars represent the standard deviation (*n* = 3). Concentrations of 3.125–50 mg/mL represent dilutions of the original extract (100 mg/mL of fibrous material in medium) with medium.

For the intended topical application of the fibrous matrices developed it is essential to ensure that they are safe to use in contact with skin and are non-toxic to cells. Therefore, cytotoxicity tests were performed in a cell line of dermal origin, namely epidermal keratinocytes (HaCaT). Cell viability was assessed by performing the MTT reduction assay in order to evaluate potential toxicity of PCL fibrous matrices, either unloaded or loaded with ACV (17%) and ω3 (33%) (Figure 7).

According to ISO 10993-5, 2009, for a medical device to be considered non-cytotoxic, it must have a viability equal to or >70% of the negative control (mock extract, i.e., medium submitted to the same treatment as test extracts). Regarding the results shown in Figure 7 it can be inferred that unloaded PCL fibrous matrix are not cytotoxic to epidermal keratinocytes cells, which is in agreement with other reported studies that confirm the safety of PCL (Prasad et al., 2015; Ravanan et al., 2016). On the contrary, PCL fibrous matrix loaded with the bioactives are cytotoxic, presumably related with the intrinsic cytotoxicity of ACV, ω3 or their combination. However, acceptable cytotoxic levels were observed for all diluted extracts (50 mg/mL or lower), which seems to indicate that drug-loaded fibers still present low toxicity potential. Indeed, it should be considered that ISO 10993-5 has been specifically developed for testing medical devices—which do not contain bioactive compound(s)—and are intended to be used as inert materials for implantation. The limited cytotoxicity shown by unloaded and drug-loaded fibrous matrices under high concentrations appears to indicate that their topical application is potentially safe.

## Discussion

Regarding other routes of administration, topical delivery of ACV in orofacial herpes lesions is the most attractive since it has excellent accessibility, low enzymatic activity, provides painless administration and permits a direct deposition of the drug at the intended site of action with a consequent reduction in the problems associated to an oral administration. These problems include low bioavailability of ACV (after oral intake only 10–20% of the drug is absorbed at the gastrointestinal level) and short plasma half-life (2.5 h) (Lopes et al., 2018). The limitations of oral administration, combined with high values of non-degraded ACV metabolites excreted through urine (9-carboxymethoxymethylguanine corresponding to 8.5–14% of the administered dose) as well as high non-specific distribution of the drug in tissues that do not constitute therapeutic target (*off-target* distribution), lead to a low drug concentration at therapeutic sites, requiring frequent administration of high doses to reach the appropriate therapeutic concentrations (Laskin, 1983). Another problem associated with the required high doses of ACV administration is the occurrence of secondary problems like phlebitis, mainly in intravenous administration at insertion site (Gurkan et al., 2012).

Despite the clear advantages of topical delivery of ACV, the commercially available formulations still have many limitations. In particular, ACV has low skin permeability and low solubility in common dosage forms such as creams, thus requiring to be administered as supersaturated formulations and in a dose that highly surpasses the required therapeutic concentration (Sponchiado et al., 2016). Also, the therapeutic scheme is very fastidious and fallible, typically requiring five applications a day, for 4 days, which sums twenty applications for the entire treatment. These applications are in some cases controlled by the dispenser of the formulation, providing 0.1 g per application with about 0.005 g of ACV. However, in other cases, commercial formulations are not available in a dose dispenser container and thus a random amount of drug is used. Notwithstanding the excess of ACV relatively to the therapeutic dose, the administrated doses of commercial topical formulations are unable to maintain therapeutically effective levels of the drug at the site of action for a long period. Local treatment often fails due to the active physiological removal mechanisms (physiological secretions and/or mechanical movements) which cause incorrect distribution of the drug in the mucocutaneous area affected by the infection, in view of the numerous applications required to maintain efficacious levels of the drug at the site of application. Moreover, the demanding therapeutic scheme proposed decreases patient compliance to the therapy. Altogether the excessive doses and the non-compliance is also regarded as a cause for possible development of resistant viruses.

To overcome the abovementioned problems and meet therapeutic requirements, formulations containing ACV which are intended for topical administration in areas affected by herpes lesions should have permanent contact with skin or mucous membranes and high resistance to physiological removal mechanisms, so as to maintain close, protracted contact between the formulation and the mucosa or epidermis affected by the herpes lesions. Other requirements include the possibility to obviate a repeated administration, providing controlled release dosage form that relieve patients from repeated applications. Unlike existing topical commercial formulations containing ACV, polymeric fibrous matrices may provide a single dosage form for this drug with all the mentioned requirements. Furthermore, despite providing the incorporation of ACV, polymeric fibers are amenable to incorporate an array of bioactives that can aid in the healing of herpes lesions. An example is ω3, which might be useful for faster healing of herpes lesions, because of its proven efficacy in the management of skin infections and wounds. In the human body, ω3 are precursors of signaling molecules that are important for: re-establishing homeostasis and resolution in chronic wounds; host defense, pain, and tissue remodeling; (Serhan, 2014) inducing anti-inflammatory and pro-resolving signaling pathways; and combating various microbes (Maderna and Godson, 2009). Additionally, in a randomized, double-blind clinical trial, ω3 application in wound care has promoted significantly faster healing while in recent case studies ω3 application has also reduced the need for use of analgesic medication (Yang et al., 2016; Dorweiler et al., 2018).

In this regard, we have successfully prepared ACV (17 wt%) and ω3 (33 wt%) loaded PCL fibrous mats using electrospinning technique. The polymeric fiber mats loaded with the bioactives exhibit a high porosity, slightly above 70%, which was smaller than the unloaded counterparts (porosity above 90%) in consequence of alterations in the shape and arrangement of the fibers upon loading (Ferreira et al., 2014). The porous structure obtained will be of high importance for the healing of herpes lesions allowing drainage of the blister exudates and permeation of atmospheric oxygen to the wound surface. Although visible by SEM, the alterations in the shape and arrangement of the fibers upon bioactives loading can be considered moderate as the fibers maintained a cylindrical morphology with average diameters of 2.2 ± 0.7 μm, being slightly larger than in unloaded PCL fibrous mats (Figure 2). Similar increase in fiber diameter upon loading of ω3 was observed in a previous study (García-Moreno et al., 2016), but in this case the fibers obtained where nanometric in diameter, either due to the smaller ω3 content (11 wt% instead of 33 wt%) or to the different polymeric composition, as fibers were made of poly(vinyl alcohol) (PVA) instead of PCL. In the literature, reports of ACV incorporation into polymeric fibers, described different polymeric composition and drug content: PAN (with 43 wt% of ACV Chen and Yu, 2010 or with 11, 25 and 43 wt% of ACV Yu et al., 2010); PVP (with 20 wt% of ACV Yu et al., 2011 or 5 wt% of ACV Baskakova et al., 2016), polyurethane, PU (with 1.2 wt% of ACV Azizi et al., 2016) and PCL (with 5 wt% of ACV Baskakova et al., 2016). All fibers revealed smooth uniform structures of nanometric size, however it is noteworthy to mention that the composition of the fibers greatly influences its morphology, as well as solvents used or electrospinning parameters. In the only case where the composition of the fiber resembles ours, the ACV content was much lower (5 wt% instead of 17 wt%) justifying the differences found in fiber diameters. Despite the larger diameters found in the fibers produced within this study, and the morphological changes imposed by the bioactives loading, we can conclude that, macroscopically (Figure 1), the loaded electrospun fibrous matrices remained smooth and quite flexible, which may offer a desirable texture to be used comfortably. The flexibility and mechanical properties of the developed fibrous matrices were further evaluated by tensile-stress studies. Indeed, since the intended use of the fibrous matrices developed is a practical application as dermal patch for orofacial herpes, the mechanical properties of the dressing are also important. Mats must be sufficiently durable for handling and application and must also withstand loads applied by the patient upon movements. Additionally, replacement of the dressing material must be carried out easily without trauma or any possible damage to renewed epithelial tissues. The mechanical properties that were determined by test tensile stress tests were elongation at break, tensile strength at break, and elastic modulus (Young' modulus). Although the mechanical properties are essential for the characterization of the polymeric fibers, no previous studies regarding fibers loaded with ACV (Chen and Yu, 2010; Yu et al., 2010, 2011; Azizi et al., 2016; Baskakova et al., 2016) or with ω3 (Moomand and Lim, 2014; García-Moreno et al., 2016) reported these properties, which would be useful for comparison purposes with our loaded fibrous matrices. Nevertheless, we can conclude that the PCL fibrous mats loaded with the bioactives demonstrated a reasonable balance between flexibility and hardness as it were able to withstand over 100% extensional strain before breaking (Table 1), a comparable value to the obtained with microfibrous commercial textile dressings such as Biofix® and Resolut LT® (Milella et al., 2001), indicating that the electrospun system can be similarly handled like existing textile products. Moreover, the Young's modulus of the fibers developed (Table 1) were in the same order as other antiviral films reported in the literature (Akil et al., 2011). For practical application as dermal patch, the flexibility of the produced loaded PCL fibrous mats will allow it to maintain close proximity to the contours of the skin surface. This flexibility is probably conferred both by PCL composition and electrospinning process of production (Ghosal et al., 2018).

The results gathered by DSC and ATR-FTIR confirmed the mechanical properties of the fibrous mat. Although, in agreement with other reports (Chen and Yu, 2010; Yu et al., 2010) a crystallinity reduction effect was observed upon the inclusion of the bioactives in the polymeric matrix; this effect was small and did not seem to compromise the mechanical resistance and structural integrity of the fiber mat. This chemical and physical characterization also provided a proof of the bioactives incorporation. Thus, in comparison to unloaded fibers, changes in DSC spectra confirmed the loading of ω3 (Supplementary Figure 4) while changes in ATR-FTIR/DSC spectra (Figure 3 and Supplementary Figure 4) and XRD diffractograms (Figure 4) confirmed the incorporation of ACV. The ATR-FTIR spectra of the ACV loaded fibers also suggested the establishment of hydrogen bonding that can occur between carbonyl groups of PCL and the hydroxyl groups of ACV (C=O…H-O) or between carbonyl groups of the polymer and amine groups of ACV (C=O…H-N) (see chemical structures in Figure 1). Indeed, upon loading of ACV in PCL fibers the attenuation and alteration of multiple peaks within 3,000–4,000 cm^−1^ region (Figure 3D) was observed, which are related with hydroxyl and amine stretching vibrations in ACV and can indicate that hydrogen bonding occurred. The same type of observations was correlated with the establishment of hydrogen bonding between ACV and the polymer PVP (Yu et al., 2011). The interactions occurring between ACV and PCL polymer within the fiber can also explain the crystallinity decrease of PCL loaded fibers observed by DSC (Supplementary Table 1) and the Young's modulus decrease observed by tensile stress studies (Table 1), as from these interactions can occur the formation of a complex polymer-drug that decreases the attraction between polymer chains to make them more flexible and more amorphous than the crystalline raw materials (Yu et al., 2010). Similarly, ω3, being an oil incorporated in the polymer matrix, could lead to polymer softening by reducing the forces between the polymer molecules, thereby increasing chain mobility (Demchuk et al., 2018; São José et al., 2019). This also justifies the crystallinity decrease of PCL loaded fibers observed by DSC (Supplementary Table 1) and the Young's modulus decrease observed by tensile stress studies (Table 1).

Besides mechanical properties, occlusion is another important parameter to be evaluated when developing a dressing for antiviral and herpes lesions healing purposes. Occlusive dressings in the context of herpes lesion can be used as immediate means of controlling the cleanliness of the lesions and to avoid viral contamination of the surrounding tissues or bacterial contamination of the lesions. Moreover, the occlusion is also related with the capacity to prevent water evaporation to provide a moist healing environment for wound sites, which simultaneously favors healing and helps to refresh the surface of skin, thereby decreasing pain and improving patient acceptability (Liu and Jia, 2018). Our loaded PCL fibrous mats showed an occlusion factor of 12% and this value was closely maintained during 108 h (Supplementary Figure 5) which represents approximately the expected duration of actual topical treatment for herpes. The commercial formulation also tested was not able to avoid water evaporation thus presenting a null occlusive factor. The occlusive factor obtained for the loaded PCL fibrous matrices was similar to the reported for a topical formulation of solid lipid nanoparticles with lower lipid content, considered to possess occlusive capacity (Souto et al., 2004). On the other hand, other skin dressings, such as hydro-films based on lactose-crosslinked fish gelatin (Etxabide et al., 2017) have shown about two times more occlusive factor (25.17 ± 0.99%) than the fibers developed in the current work. This higher value of occlusive factor obtained is understandable considering that these reported systems are films (thus made of continuous material) and not fibrous porous nets like our system. Moreover, we should highlight that these authors have performed the occlusive assay at room temperature (20°C) while we have processed the same assay at a controlled temperature of 37°C (simulating the elevated skin temperature resulting from inflammation), thus increasing the water evaporation losses. Finally, it is noteworthy to mention that the value of WVTR found for PCL loaded matrices was 881 ± 91 g/m^2^day, which is in the range of commercial skin dressings (426–2,047 g/m^2^ day) (Tu et al., 2015).

In herpes treatment, a sustained release of antiviral agents is desirable for maintaining the drug at a sufficient concentration and long enough for the intended pharmaceutical effect to occur, additionally providing extended periods of coverage that may increase patient adherence to therapy. The polymeric fiber mats loaded with ACV and ω3 were found to fulfill this requirement when analyzed for *in vitro* ACV release. PCL fibrous matrices provided a sustained and complete ACV release profile in which 54% of the drug is released in 24 h while the total drug content is released in about 96 h (Figure 5A). The ACV release from the fibers was slower (Supplementary Table 5) and more complete than from the commercial formulation which appears to be due to the large surface area of the fibers permitting a wider dispersion of ACV on the fiber which facilitates a more complete diffusion of the drug into the dissolution medium. In terms of concentrations, the cumulative release of ACV from the fibers was ~9 μM at the first 30 min, 140 μM at 24 h and 260 μM over the total 103 h of the assay. Thus, the 17 wt% of ACV loaded in the fibers assured that at all time points the concentration of ACV was always superior to the required to block HSV replication *in vitro* (0.16–3.77 μM) (Sponchiado et al., 2016). Furthermore, if we consider the fibrous mat as a patch to be applied during a 24 h period, by this time the ACV released is 27 folds greater than the highest IC_50_ reported, which may guarantee a successful therapeutic efficacy within a shorter period (1 day instead of 4 days) and with a dose that is at least 5 times lower that the supplied by the commercial formulations.

The PCL fiber mats loaded with ACV and ω3 were also found to have a superior performance than other ACV loaded fibers reported in the literature when analyzed for *in vitro* ACV release (Table 3).

**Table 3:** Information regarding ACV release from polymeric fibers.

**Polymeric fiber composition**	**ACV wt%**	**ACV released in the first hour (%)**	**ACV total release (%)/Time**	**Reference**
PVP	20	n.d.	100/1 min	Yu et al., 2011
PVP	5	n.d.	71/23 h	Baskakova et al., 2016
PCL	5	n.d.	82/88 h	Baskakova et al., 2016
PAN	43	40	89/16 h	Chen and Yu, 2010
PAN	11	31	63/36 h	Yu et al., 2010
PAN	25	34	67/36 h	Yu et al., 2010
PAN	43	53	88/36 h	Yu et al., 2010
PU	1.2	≈50	75/70 h	Azizi et al., 2016
PCL	11	5	100/96 h	Current study

The data summarized in Table 3 indicate that PCL fibers provide a more complete release of ACV while prolonging the release of ACV over 4 days (96 h) which seems to be an ideal treatment period for herpes. Besides the evident differences in fiber composition and drug loading that might affect the release behavior, the initial inner structure and the dispersion pattern of drug within the polymer matrix should be considered as critical factors controlling the release process. PCL loaded fibers developed in this work have larger diameters than the fibers enumerated in Table 3 which may retard the release medium penetration within the fibers mesh. Accordingly, another study has shown that loading a highly water-soluble compound (acetaminophen) in hydrophobic poly (_DL_-lactide) fibers led to slower release of the drug with an increase of fiber diameters from 200 nm to 1.5 μm (Cui et al., 2006). Furthermore, according to static water contact angles (Supplementary Figure 1B) ACV seems to be loaded in the fibers core and thus its release occurs very slowly (as confirmed for the release obtained in the first hour, Table 3). This may also explain the sustained release profiles obtained.

The skin permeation of ACV was also evaluated and agreed with the observed sustained drug release profile, previously described. Indeed, when compared with the commercial formulation or other reported polymeric fibers (Yu et al., 2011), PCL fibrous matrix retarded the flux rate of drug across the skin. In terms of permeability PCL fibrous matrices were able to improve the drug permeation up to 21 times, when comparing to permeation achieved by non-formulated free ACV (Lopes et al., 2018). The higher permeation of ACV loaded in PCL fibrous mats, over the free drug and over ACV loaded in the commercial formulation, may be attributed to the solubility improvement of ACV due to molecular dispersion within PCL and to enormous increase in the surface area provided by fibers mats produced by electrospinning.

In addition to releasing and permeation of bioactives, it is also critical that fibers are safe in biological systems. The toxicity of fibers was assessed using a MTT viability assay in epidermal keratinocytes cells according to ISO 10993-5 (2009) guidelines for biological evaluation of medical devices for implantation. Cells exposed to PCL fibrous matrix loaded with the bioactives presented overall low cytotoxicity. Interference with cell viability was only apparent for high concentrations of ACV and/or ω3. Still, it should be noted that the amounts of fibers (and, consequently, drugs) used in this *in vitro* setting is much higher than the predictable amounts that will be used *in vivo*. Thus, presented data appears to sustain that the proposed fibrous matrices are safe for topical application.

As with any other research this study has some limitations like the need to further explore, in a follow-up investigation, the skin permeation and therapeutic effect (e.g., by studying the anti-inflammatory effect that favors the healing process) of the ω3 incorporated into the fibers, as well as the oxidative stability of this component during storage. Up to this point we have relied in the literature reports that reveal the cosmetic and therapeutic benefits of topical application of ω3 in skin (Huang et al., 2018) and assure that fibers provide a greater oxidative stability to this bioactives (Moomand and Lim, 2014). Other planned assays are skin cellular adhesion and proliferation studies and cytotoxicity tests by direct contact of the cells with the fibers.

In conclusion, there is a great need to develop multipurpose topical strategies that provide antiviral therapeutic efficiency in addition to delivering bioactive compounds capable of promoting faster healing of the herpes blisters. To our knowledge, this research represents the first electrospun fibrous matrices proposed as dressings for application in orofacial herpes. The combination of bioactives offered by our fibers were not proposed by any other study reported. Topical application of the loaded fibrous matrices developed would address an important gap in commercially available topical formulations, such as the fastidious therapeutic regimens and the lack of patient compliance, as well as ultra-excessive antiviral drug doses. We envision that a skin patch (2 × 2 cm) made of the PCL fibrous matrix developed, would provide ACV release with several advantages toward the current formulations: (i) sustained drug release, permitting a continuous treatment for at least 24 h with a dose that assures efficacy in arresting virus replication (27 folds greater than the highest IC_50_ reported) and that is at least 5 times lower that the current regimens; (ii) high porosity (> 70%) and mild occlusion (12%), indispensable for faster healing; (iii) greater patient compliance and control of the therapeutic doses.

## Data Availability Statement

All datasets generated for this study are included in the article/Supplementary Material.

## Author Contributions

ML and TV contributed to the conception and design of the study. TC performed most of the experimental work, including the preparation and characterization of fibers, and analysis of results. SL-M and CR performed DSC and contact angle experiments. RM performed ATR-FTIR assays. AR performed permeability skin studies and AC-P provided the necessary conditions for this assay. AA and JN performed cellular toxicity assays. TV wrote the first draft of the manuscript. AR, TC, TV, and ML wrote sections of the manuscript. ML wrote the final version of the manuscript. All authors contributed to manuscript revision, read, and approved the submitted version.

### Conflict of Interest

The authors declare that the research was conducted in the absence of any commercial or financial relationships that could be construed as a potential conflict of interest.
