# Supplementary material for: Polymeric Electrospun Fibrous Dressings for Topical Co-delivery of Acyclovir and Omega-3 Fatty Acids
Source: Front Bioeng Biotechnol. 2019 Dec 3;7:390. doi: 10.3389/fbioe.2019.00390 (PMC6901623; doi:10.3389/fbioe.2019.00390)
Supplement: Supplementary file 1 [file Data_Sheet_1.PDF]

Supplementary Material

1 Measurement of polymeric fibrous matrices thickness and static water contact angle

The thickness of the fibrous mats was accessed by cutting a frozen piece of fiber in liquid nitrogen with a micrometric scalpel to avoid deformation at the cutting edge and analyzing the edge by SEM (Figure S1A). The static water contact angles (Figure S1B) were also measured for analyzing surface hydrophilicity of the fibrous mats.

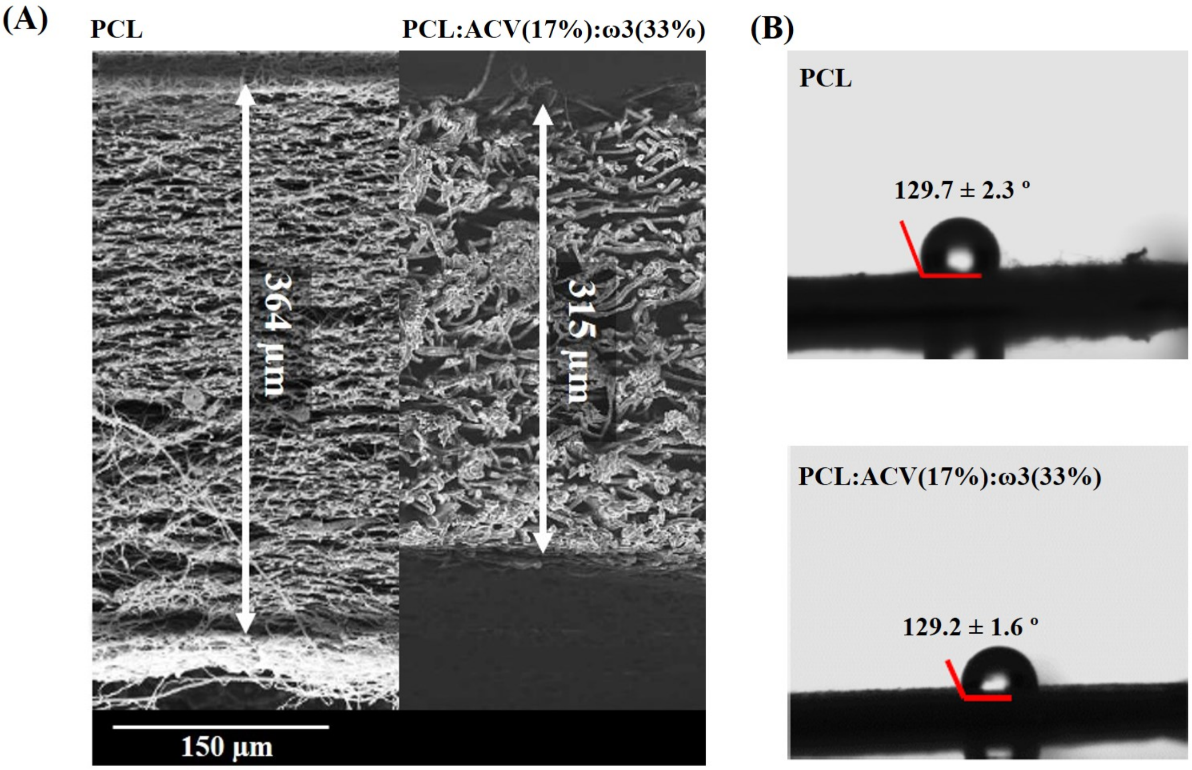

Supplementary Figure 1. (A) SEM image of the edge of the fibrous matrix unloaded (PCL) and loaded with the bioactives (PCL:ACV(17%):ω3(33%)); **(B)** Micrograph of static contact angle of a water droplet in the surface of the fibrous matrix unloaded (PCL) and loaded with the bioactives (PCL:ACV(17%):ω3(33%)).

2 Measurement of stress-strain mechanical response

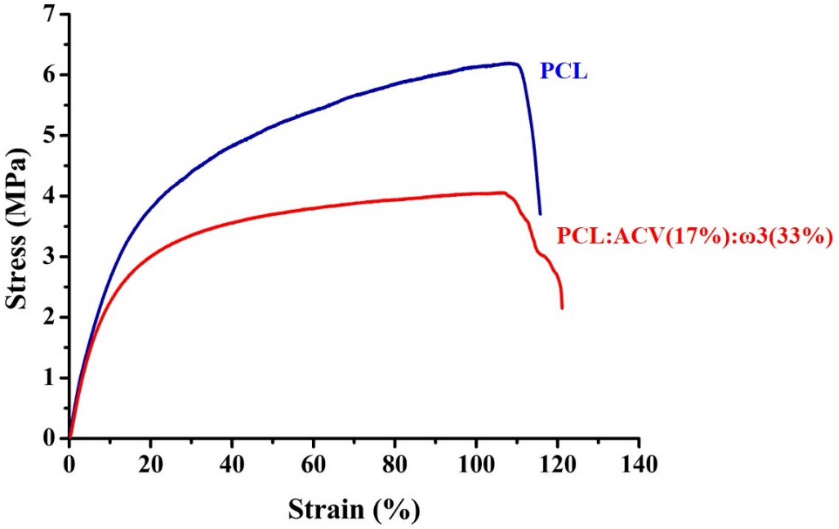

Supplementary Figure 2. Typical stress–strain curves for PCL fibrous matrix unloaded (PCL) and loaded with the bioactives (PCL:ACV(17%):ω3(33%)).

3 Chemical characterization by ATR-FTIR

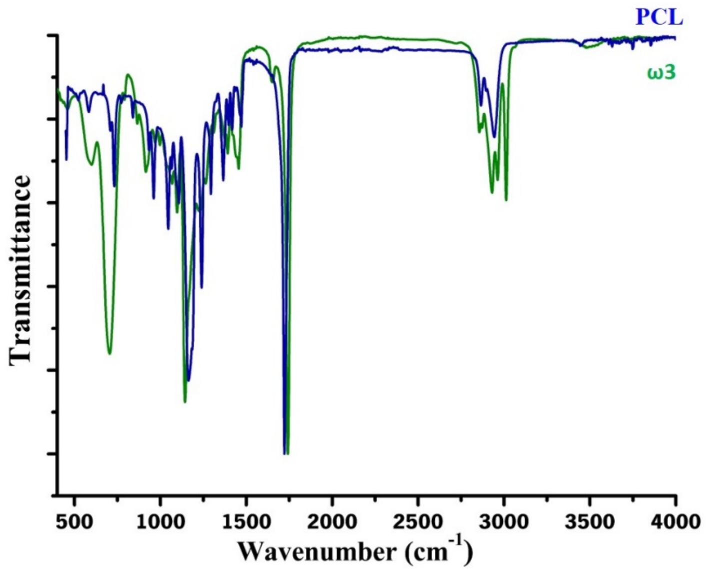

Supplementary Figure 3. ATR-FTIR spectra for PCL fibrous matrix unloaded (PCL, blue) and for ω3 fatty acids (ω3, green), where it is observed the superimposition of most of the characteristic vibrational bands.

4 Thermodynamic characterization by DSC

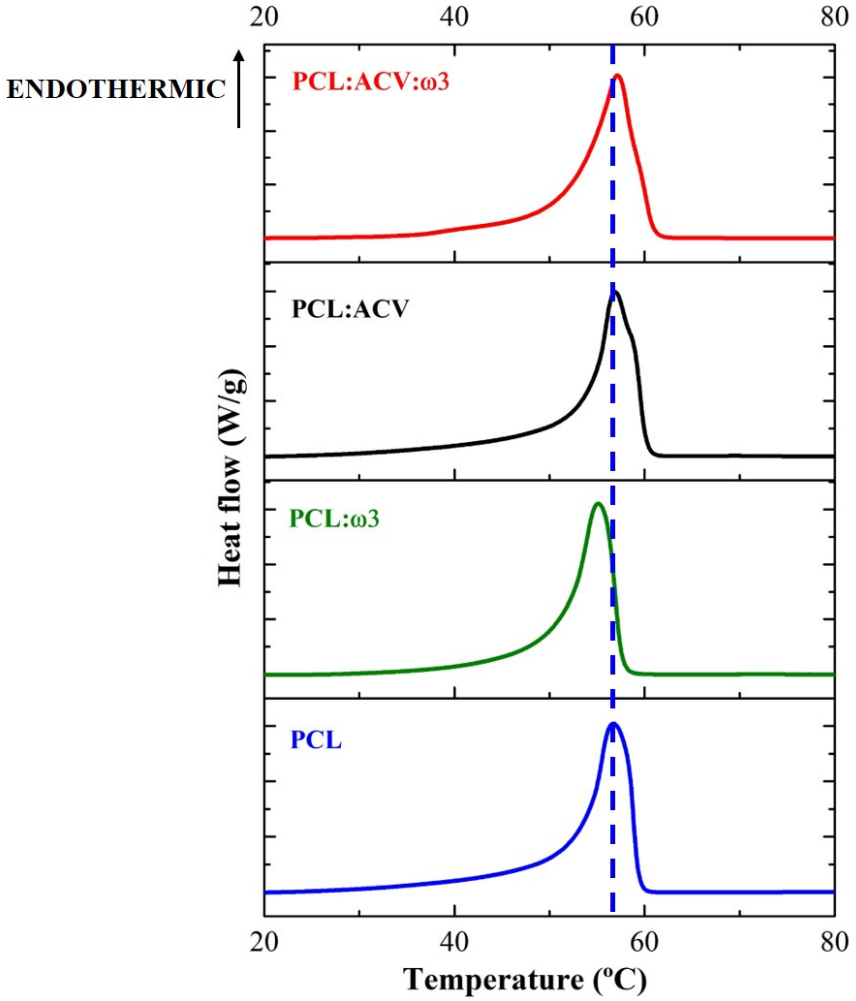

Supplementary Figure 4. DSC thermograms for PCL fibrous matrix unloaded (PCL, blue) and for PCL fibrous matrices loaded with: ω3 (green), ACV (black) and ACV (17%)+ω3(33%) (red). Dashed line is a guideline to observe each peak position relatively to the endothermic melting peak of PCL.

Supplementary Table 1. Thermodynamic parameters obtained from the DSC characteristic thermograms of PCL fibrous matrix loaded with: ω3 (PCL:ω3) and ACV (PCL:ACV).

	T_f (°C)	ΔH_f (J/g)	*Crystallinity loss (%)* [(a)]
PCL:ACV	56.9	38.2	32.1
PCL:ω3	55.1	35.7	36.6

[(a)] $Crystallinity\ loss\ (\%) = \frac{\Delta H_{PCL\ fibers} - \Delta H_{PCL\ loaded\ fibers}}{\Delta H_{PCL\ fibers}} \times 100$

5 Determination of the occlusive factor

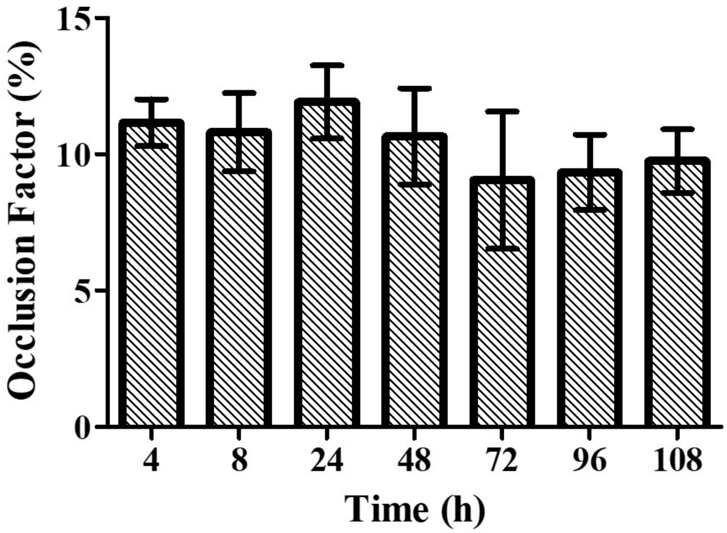

Supplementary Figure 5. Occlusion factor of PCL fibrous matrices loaded with 17 wt% of ACV and 33 wt% of ω3.

6 Mathematical modelling for the description of the drug release profile

Supplementary Tables 2 to 5 – Fitting of ACV release from PCL fibers [PCL:ACV(17%):ω3(33%)] in micellar medium (37 ºC at pH 5.5).

Table S2 - 1st Order kinetics

Mathematical equation		Adjusted parameters	R^2	R^2 adjusted
$F_{max}(1 - e^{-kt})$	PCL:ACV(17%): ω3(33%) fiber	F_{max}= 104.50±1.59<br>k = 0.033±0.001	0.9963	0.99611
	Zovirax™ cream	F_{max}= 65.62±2.16<br>k = 0.091±0.011	0.9595	0.95725

F_{max} is the total amount of drug released and k is the first order release constant.

Table S3 - Higuchi Model

Mathematical equation	pH	Adjusted parameters	R^2	R^2 adjusted
$kt^{0,5}$	PCL:ACV(17%): ω3(33%) fiber	k =10.85±0.26	0.9651	0.9651
	Zovirax™ cream	k =10.14±0.14	0.9852	0.9852

k is the Higuchi dissolution constant.

Table S4 - Korsmeyer–Peppas Model

Mathematical equation		Adjusted parameters	R^2	R^2 adjusted
at^n	PCL:ACV(17%): ω3(33%) fiber	a = 10.56±1.71<br>n = 0.506±0.0387	0.9652	0.96337
	Zovirax™ cream	a = 13.05±0.46<br>n = 0.4287±0.0099	0.9959	0.99567

$\boldsymbol{a}$ is a constant of geometric and structural incorporation that takes into account the pharmaceutical form; n is a release index representing the mechanism of diffusion of the drug, being based on Fick's law, (a value of n equal to or less than 0.5 indicates a Fickian diffusion, whereas values between 0.5 and 1 indicate a non-Fickian diffusion.

Table S5 - Gallagher-Corrigan Model

Mathematical equation		Adjusted parameters	R^2	R^2 adjusted
$Fb\,(1-e^{-k_1 t}) + (F_{max}-Fb)\left(\frac{e^{-k_2 t - k_2 t_{max}}}{1+e^{-k_2 t - k_2 t_{max}}}\right)$	PCL:ACV(17%): ω3(33%) fiber	F_b = 84.44±14.93<br>F_{max} = 101.80±2.75<br>t_{max} = 36.29±1.515<br>k_1 = 0.0391±0.0103<br>k_2 =0.0767±0.0231	0.9968	0.99600
	Zovirax™ cream	F_b = 27.31±3.20<br>F_{max} =70.4700±1.37<br>t_{max} = 22.54±1.45<br>k_1 = 0.333±0.077<br>k_2 = 0.118±0.019	0.9955	0.99430

$\boldsymbol{F_b}$ is the amount of drug released directly from surface of the system (initial burst), $\boldsymbol{F_{max}}$ is the amount of drug released during the process, $\boldsymbol{t_{max}}$ is the time (in hours) in which occurs the release of maximum amount of drug directly from the surface of system (after burst), $\boldsymbol{k_1}$ and $\boldsymbol{k_2}$ are constants of release *(h^{-1})* of first and second phase, respectively.
